# Bouveret’s syndrome—a rare form of gallstone ileus causing death: appearance on post-mortem CT and MRI

**DOI:** 10.1259/bjrcr.20170032

**Published:** 2017-05-20

**Authors:** Silvio Giancarlo Bruni, Michael Pickup, Dawn Thorpe

**Affiliations:** ^1^Department of Medical Imaging, University of Toronto, Toronto, ON, Canada; ^2^Department of Laboratory Medicine and Pathology, University of Toronto, Toronto, ON, Canada; ^3^Provincial Forensic Pathology Unit, Ontario Forensic Pathology Service, Toronto, ON, Canada

## Abstract

Bouveret’s syndrome is a very rare cause of gastric outlet obstruction occurring as a complication of cholelithiasis with cholecystogastric or cholecystoenteric fistula. Without timely diagnosis and intervention Bouveret’s syndrome can be associated with a high rate of morbidity and mortality, with common causes of death including metabolic derangements, aspiration pneumonia and post-operative complications. We report the case of a 67-year-old female found dead in her home with regurgitated gastric contents filling her mouth and nasal cavity. Post-mortem CT and MRI imaging was performed and subsequently revealed a fistulous tract between the gallbladder and proximal duodenum, with impaction of a large obstructing stone in the mid-portion of the duodenum. Post-mortem imaging also revealed findings of gastric outlet obstruction, which was presumed to be a primary contributor to her cause of death.

## Background

Although spontaneous internal biliary fistulas occur frequently in cases of biliary calculi, obstructive gallstone ileus remains a relatively rare complication, occurring in only approximately 0.3–4% of all cases of chronic cholecystitis.^1,2^ Obstructions secondary to biliary fistula typically occur when gallstones exceed 2 cm in diameter, with a vast majority occurring in the terminal or distal ileum (60–70%).^3^ Other locations of obstruction are much rarer, with cases of duodenal obstruction accounting for only 1–3% of all cases of gallstones causing gastrointestinal obstruction.^1,4^ This particular subset, also known as Bouveret’s syndrome, occurs when a gallstone passes via a cholecystoduodenal or choledochoduodenal fistula into duodenum results in gastric outlet obstruction. This rare condition, first described in 1896 by Leon Bouveret, most frequently occurs in elderly females, and is consequently associated with a relatively high rate of mortality.

Here we present a post-mortem case of Bouveret’s syndrome leading to gastric outlet obstruction and death.

## Materials and methods

### Case history

This 68-year-old female died suddenly at home. She had been complaining of abdominal pain and intermittent vomiting for 2 days prior to her death. She infrequently visited physicians, and did not seek medical care for this illness. Her medical history obtained from family members included hypertension, asthma, chronic pain and “stomach problems”. She reportedly took opioid medications on a regular basis.

#### External examination and sample collection

External examination was performed prior to post-mortem CT (PMCT) and post-mortem MR imaging (PMMR).

#### Imaging

Before MRI examination, the body was examined with unenhanced PMCT. PMCT imaging was performed using a GE Healthcare 64-slice Optima CT660 (GE Healthcare, Toronto, ON) kVp 140, mA 185, helical slice thickness 1.25, Pitch 0.51:1, Speed 20.62 mm rot^–1^, Rotation time 0.5 (s). The time interval between death and initial PMCT imaging was approximately 20 h.

Post-mortem MRI was subsequently performed using a GE Healthcare 1.5 Tesla 450 W wide bore MRI unit (GE Healthcare, Toronto, ON) and imaged with use of a GEHC 12-channel Body Array coil. Standard axial and coronal *T*_2_ weighted, with (TE: 80ms, TR: 3586/2872 ms, 4 mm thickness) and without (TE: 80 ms, TR: 4025/2800 ms, 3 mm thickness) fat saturation, axial and coronal *T*_1_ weighted (TE: 9/10 ms, TR: 550 ms, 4 mm thickness), and sagittal *T*_2_ weighted 3D CUBE fast spin echo images with axial and coronal reconstructions were acquired through the abdomen and pelvis. Examination time was 45 min.

PMCT and PMMR Images were evaluated using the GE Centricity Universal (DICOM) viewer.

#### Internal examination and sample collection

Complete pathologic internal examination was performed subsequent to imaging. This included gross and histologic examination of the organs and tissues with particular attention to pathologic findings. Histologic sections of brain, heart, lung, liver, kidney, bile duct and gallbladder were stained with hematoxylin and eosin and examined by a forensic pathologist using light microscopy. Blood samples (from iliac vessels) and vitreous fluid were collected by needle aspiration. Given the history of opioid use, routine toxicologic analysis of post-mortem blood and urine was also performed by the Centre of Forensic Sciences in Toronto.

## Results

### PMCT and PMMR

**Figure 1.**
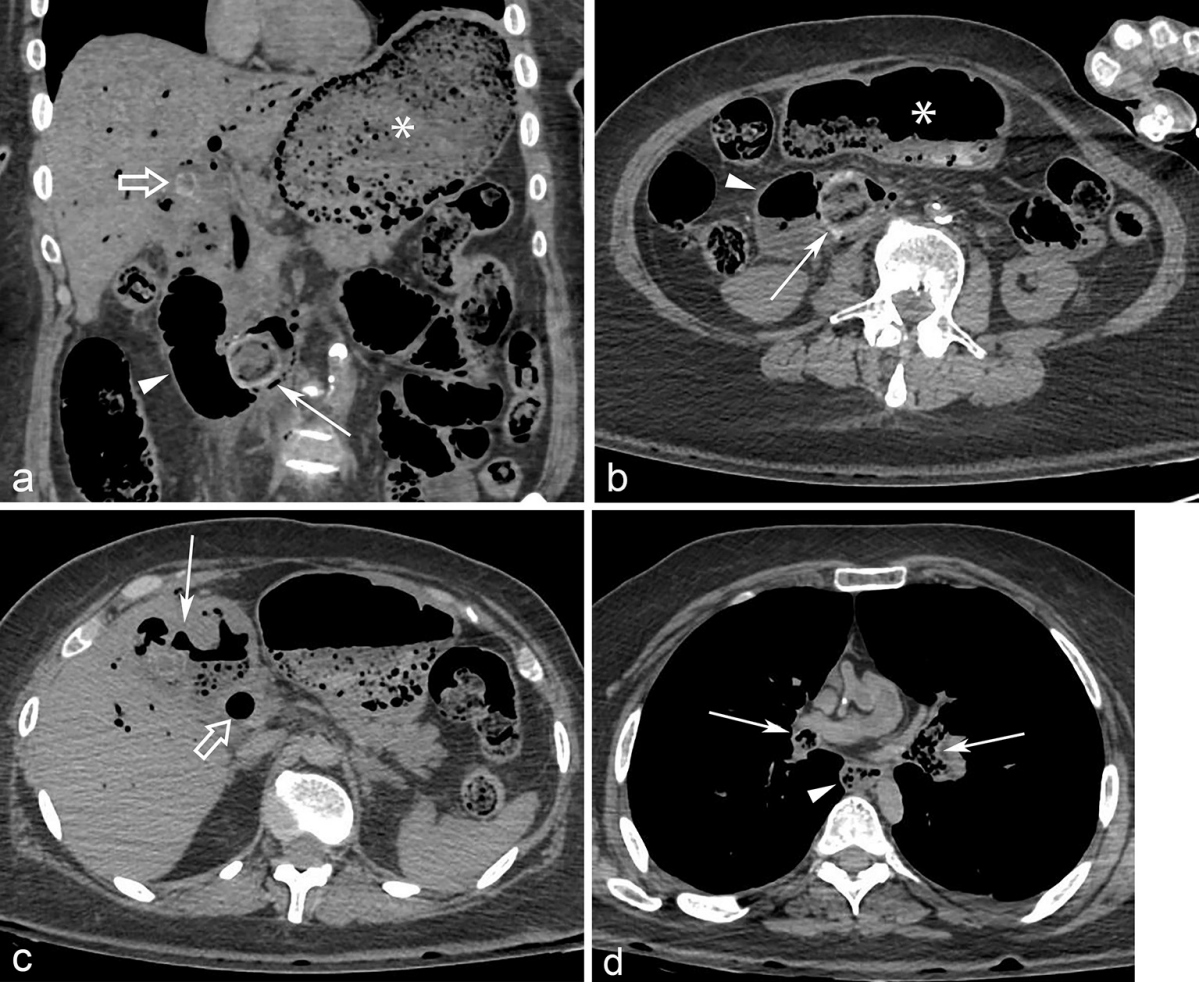
Post-mortem unenhanced CT in case of Bouveret’s syndrome. Coronal (a) and axial (b) unenhanced CT images through the level of the upper abdomen demonstrate a markedly distended stomach (asterisks) and proximal duodenum (arrowheads). A large peripherally calcified gallstone is seen impacted in the 4th part of the duodenum (arrow). Several other calcified calculi are also seen within the gallbladder (open arrow), with extensive pneumobilia throughout the intrahepatic biliary tree. Axial (c) image at the level of the gallbladder shows a gas and fluid communication (arrow) between the gallbladder fundus and adjacent duodenal bulb. The pancreatic portion of the common bile duct is dilated by gas (open arrow). Axial (d) image in the same patient at the level of the main bronchi demonstrates mixed density material throughout the central tracheobronchial tree (arrows). Debris extends into the second and third order branches (not shown), and is also seen filling the esophagus (arrowhead).

Unenhanced PMCT of the thorax demonstrated a large volume of mixed density debris filling the tracheobronchial tree, with only minimal ground glass opacity in the dependent lungs. Unenhanced PMCT of the abdomen and pelvis was also performed, which showed a massively distended stomach filled with mixed density ingested material (Figure 1). More inferiorly, a peripherally calcified gallstone was seen impacted in the proximal fourth part of the duodenum. Multiple other large calculi and gas were also seen within the thickened gallbladder. A thick-walled tubular gas-filled structure was demonstrated, extending from the gallbladder fundus to the duodenal bulb, presumed to represent a widely patent fistulous tract. Extensive pneumobilia was also seen throughout the extra- and intrahepatic biliary tree. No other definitive pathologic abnormality was seen.

**Figure 2.**
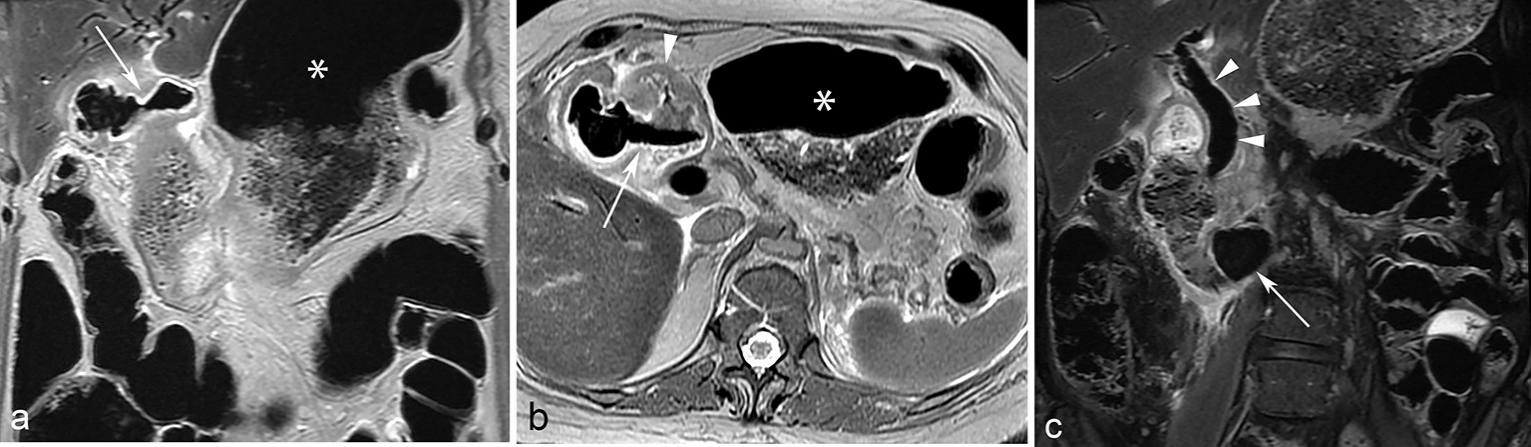
Post-mortem unenhanced MRI in case of Bouveret’s syndrome. Coronal (a) and axial (b) *T*_2_ weighted images through the level of the gallbladder fossa clearly demonstrate a widely patent gas-filled fistula (arrow) extending from the gallbladder to the adjacent duodenal bulb (arrow), just beyond the pylorus (arrowhead). The fistula is peripherally lined with hyperintense fluid. Debris can be seen filling and distending the stomach (asterisk). Coronal *T*_2_ weighted fat suppressed (c) image demonstrates a large signal void representing the obstructing duodenal gallstone (arrow), partially surrounded by hyperintense fluid in the dilated proximal duodenum. The extrahepatic bile duct (arrowheads) is dilated and gas-filled.

At PMMR, the widely patent cholecystoduodenal fistula was seen to a much greater advantage on *T*_1_ and *T*_2_ weighted axial and coronal sequences (Figure 2). *T*_1_ weighted sequences also again demonstrated an impacted calculus within the proximal fourth portion of the duodenum, with dilatation of the duodenum proximal to this, as well as massive gastric distention. On both *T*_1_ and *T*_2_ weighted images, the biliary tree appeared predominantly gas-filled, without definite visualization of intraductal calculi.

#### Autopsy and other investigations

External examination revealed an overweight female (BMI 28 kg m^–3^) without evidence of injury. Coffee-ground type material was present on the face emanating from the nose and mouth. The abdomen was not distended, and there were no external signs of putrefaction.

Internal examination revealed features of chronic cholecystitis with a markedly thickened gall bladder wall, dense fibrous tissue deposition in the gallbladder bed with extension into the pericholecystic hepatic parenchyma and adjacent pancreas and duodenum. A small quantity of “changed” blood was present in the stomach. There was no evidence of peptic ulcer disease, or haemorrhagic gastropathy.

**Figure 3.**
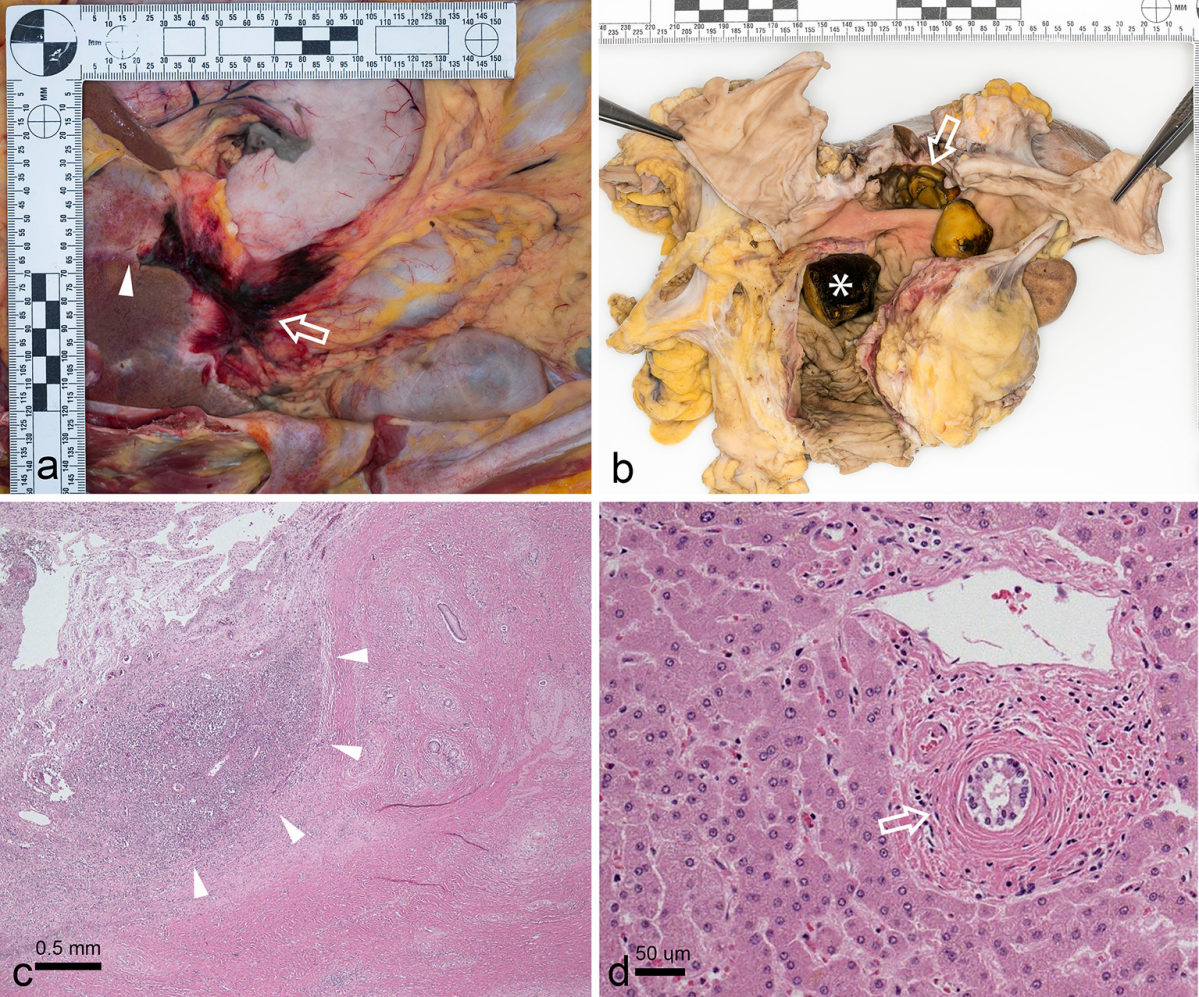
Gross and microscopic photographs from autopsy. (a) Open peritoneum demonstrating gallbladder bed fibrosis extending into the pericholecystic hepatic parenchyma (arrowhead). Fibrosis and acute haemorrhage (thick arrow) also extends from the gallbladder fossa to the adjacent gastric antrum. (b) En bloc resection of the duodenum, gallbladder, pancreatic head and part of the liver demonstrates multiple faceted calculi filling the gallbladder (thick arrow). The gallbladder wall appears markedly thickened and fibrotic. A widely patent epithelialized fistula fistulous tract between the gallbladder and duodenal bulb is partially obscured by a large calculus within the proximal duodenum, at the level of the fistula (arrowhead). A larger 3 cm faceted calculus is seen more distally (asterisk) and was impacted at the junction of the 3rd and 4th part of the duodenum, with staining of the duodenal mucosa at the site of impaction (red arrow). (c) Histologic (H&E) section of gallbladder bed showing focus of acute cholecystitis (black arrowheads) and chronic fibrous tissue deposition (asterisk). (d) Histologic (H&E) section of intra-hepatic portal triad with “onion-skin” fibrosis surrounding bile duct indicative of (secondary) sclerosing cholangitis.

Upon dissection of this area, a large cholecystoduodenal fistula, and impacted duodenal gallstone were identified (Figure 3). This was associated with a small amount of acute haemorrhage. Histologically, there was marked acute and chronic cholecystitis with secondary sclerosing cholangitis involving the intrahepatic bile ducts. Ascending cholangitis was not identified.

Histologic examination of the lungs revealed aspirated gastric debris without a vital reaction. There was no evidence of aspiration pneumonia or pneumonitis. The background lung parenchyma showed emphysematous change. The heart showed microscopic hypertensive changes (cardiomyocyte hypertrophy and interstitial fibrous tissue deposition). Changes related to chronic hypertension were also seen in the kidneys. Analysis of the blood and urine was negative for drugs and toxins.

#### Medicolegal conclusions

Based on the post-mortem examination, the cause of death was determined to be gallstone ileus with cholecystoduodenal fistula complicating acute on chronic cholecystitis due to cholelithiasis.

The precise mechanism of death is unclear, but may be related to fluid and electrolyte abnormalities resulting from the small bowel obstruction in combination with comorbid hypertensive heart disease and emphysema.

## Discussion

Bouveret’s syndrome is an exceedingly rare complication of cholelithiasis in which a gallstone becomes impacted within the distal stomach or proximal duodenum. Although gallstone ileus can occur in as many as 15% of patients with cholecystoenteric fistula, impaction of a bile duct stone in the upper gastrointestinal tract occurs in only 3% of these cases, often when stones measure 2.5 cm or larger.^4,5^ Gallstone ileus demonstrates a female predominance, and is 7 times more common in patients over 70 years of age, often with several comorbidities.^6^ The clinical presentation can be relatively nonspecific, including epigastric and right upper quadrant pain, nausea, vomiting, fever, dehydration and weight loss. Infrequently, in cases of duodenal or celiac artery erosion, Bouveret’s syndrome may also present with haematemesis.^2,7–9^

Given the advanced age of patients and frequency of concomitant disease including significant pulmonary disease, cardiac disease and diabetes,^10^ timely diagnosis and surgical or endoscopic intervention for stone extraction and alleviation of the gastric outlet obstruction are essential. Despite significant improvements in the early diagnosis and treatment of this condition, morbidity and mortality rates remain high, estimated at 60% and 12–30%, respectively.^1,11,12^ Delay in seeking medical attention after intestinal obstruction is common, and up to 40% of patients with gallstone ileus have no known history of hepatobiliary disease, so that even once medical help is sought there may be a significant delay in diagnosis. Common causes of morbidity include metabolic derangements precipitated by acute gastric outflow obstruction,^13^ aspiration pneumonia,^9,14^ and post-operative complications.^8,9,15,16^

Although several cases of Bouveret’s syndrome causing death are reported in the literature, to the best of our knowledge, this is the first instance of time post-mortem imaging has been employed in the diagnosis of Bouveret’s syndrome leading to death. Although diagnosis has historically been made by endoscopy, non- invasive imaging, including both CT and magnetic resonance cholangiopancreatography (MRCP) have been increasingly utilized in the pre-surgical diagnosis of this rare pathology.

Plain abdominal radiography is generally of low diagnostic value and relatively non-specific in cases of Bouveret’s syndrome. Findings of bowel obstruction, pneumobilia and an ectopic calcified gallstone (i.e. Rigler’s triad) may suggest the diagnosis in 10–50% of cases.^17–19^ Dual air-fluid levels in the right upper quadrant, representing the dilated stomach and air in the gallbladder, may also suggest the presence of a biliary-enteric fistula. Plain film may be used to follow the migration of a stone or stone fragments following lithotomy.

Similarly, in many cases ultrasound may demonstrate findings suggestive of Bouveret’s syndrome such as pneumobilia and gastric distention, but frequently can be technically difficult to perform and interpret, due to intestinal distension, collapse or air within the gallbladder. A large obstructing gallstone can often be detected by ultrasound; however the precise location (i.e. whether ectopic or orthotopic) of the stone may be difficult to establish, particularly when the gallbladder is decompressed or contracted.^20–22^ Similarly, the biliary-enteric fistula may be directly visualized on ultrasound, but may be confused with the common bile duct if not considered at the time of the study.^3^

On CT, findings of Rigler’s triad are often readily apparent and is virtually pathognomonic for Bouveret’s syndrome. CT alone is diagnostic in approximately 60% of cases,^3,19,23,24^ and can provide additional information with regards to the degree of obstruction and suggested site of fistula formation. However, in 15–25% of cases, the obstructing gallstone is isoattenuting to, and may be consequently obscured by surrounding fluid or bile within the duodenum.^3^ Oral contrast agents can significantly improve the diagnostic sensitivity of CT by both surrounding the stone within the gastrointestinal tract, as well as enhancing the tract of the biliary-enteric fistula and gallbladder lumen.

In cases where a gallstone is isoattenuating relative to bile or fluid, and oral contrast material cannot be tolerated (as in our case), MRCP or other *T*_2_ weight MR sequences may serve as a useful adjunct for confirming the diagnosis of gallstone ileus or better delineating fistula anatomy.^3,20,25^ On MRCP ectopic calculi will appear as signal voids, allowing them to be easily differentiated from surrounding hyperintense fluid within the gastrointestinal tract. Further, MRCP may depict small amounts of fluid within a collapsed fistulous tract that may be otherwise difficult or impossible to appreciate on CT alone.

Endoscopic treatment is often considered the first-line therapeutic option for Bouveret’s syndrome in patients at high risk for perioperative complications, despite having a low reported success rate (approximately 10%). In general, the success rate of endoscopic extraction is largely dependent on stone size, with stones larger than 2.5 cm much more difficult to extract endoscopically.^1,10,12^ For larger stones, endoscopic extraction may be supplemented by endoscopic laser lithotripsy, extracorporeal shockwave lithotripsy and intracorporeal electrohydraulic lithotripsy.^24^ Surgery is nevertheless needed in up to 90% of patients. In patients with significant comorbidities or in critical condition, a two-stage procedure is often performed, with urgent enterolithotomy performed initially to resolve the gastric outlet obstruction, followed by cholecystectomy and fistula closure performed at a later date. Both procedures are typically performed with open laparotomy, although laparoscopic surgery can be performed in select cases.

In summary, Bouveret’s syndrome is an extremely rare complication of cholelithiasis and biliary fistula, which as evidenced by the presented case, can result in significant morbidity and mortality. In our case, the duodenum at the level of the obstructing gallstone and the fistulous tract were both sufficiently gas-filled to allow for easy identification on unenhanced CT. However, both the obstructing calculus and fistula were depicted to far greater advantage on MR images, highlighting the utility of this modality in cases where CT is equivocal and oral and/or IV contrast material cannot be tolerated.

## Learning points

1. Due to advanced age and increased frequency of concomitant disease, Bouveret’s syndrome is associated with a high rate of mortality, and timely diagnosis and stone extraction for alleviation of the gastric outlet obstruction is essential.
2. Radiographic or CT demonstration of Rigler’s triad (bowel obstruction, pneumobilia and an ectopic calcified gallstone) is virtually pathognomonic for Bouveret’s syndrome.
3. In cases where a gallstone is isoattenuating relative to bile or fluid, and oral contrast material cannot be tolerated, MRCP or other *T*_2_ weight MR sequences may serve as a useful adjunct for confirming the diagnosis of gallstone ileus or better delineating fistula anatomy.

## Consent

Informed consent was obtained from all individual participants included in the study, or their Power of Attorney for Personal Care for publication of this case report, including accompanying images.
